# Purslane (Portulaca oleracea) Seed Consumption And Aerobic Training Improves Biomarkers Associated with Atherosclerosis in Women with Type 2 Diabetes (T2D)

**DOI:** 10.1038/srep37819

**Published:** 2016-12-05

**Authors:** Firouzeh Dehghan, Rahman Soori, Khadijeh Gholami, Mitra Abolmaesoomi, Ashril Yusof, Sekaran Muniandy, Sara Heidarzadeh, Parvin Farzanegi, Mohammad Ali azarbayjani

**Affiliations:** 1 Department of Exercise Physiology, Faculty of Physical Education and Sport Sciences, University of Tehran, Tehran, Iran; 2 Department of Physiology, Faculty of Medicine, University of Malaya, 50603 Kuala Lumpur, Malaysia; 3 Department of Molecular Medicine, Faculty of Medicine, University of Malaya, 50603 Kuala Lumpur, Malaysia; 4 Department of Exercise Science, Sports Centre, University of Malaya, 50603 Kuala lumpur, Malaysia; 5 Department of Exercise Physiology, Central Tehran Branch, Islamic Azad University, Tehran, Iran; 6 Department of Exercise Physiology, Sari Branch, Islamic Azad University, Sari, Iran

## Abstract

The aim of this study was to investigate the responses of atherosclerosis plaque biomarkers to purslane seed consumption and aerobic training in women with T2D. 196 women with T2D were assigned into; (1) placebo (PL), (2) aerobic training+placebo (AT + PL), 3) purslane seeds (PS), aerobic training+purslane seeds (AT + PS). The training program and purslane seeds consumption (2.5 g lunch and 5 g dinner) were carried out for 16 weeks. The components of purslane seed were identified and quantified by GC–MS. Blood samples were withdrawn via venipuncture to examine blood glucose, low-density lipoprotein (LDL), high-density lipoprotein (HDL), cholesterol, triglycerides (TG), creatinine, urea, uric acid, NF-κB, GLP1, GLP1R, TIMP-1, MMP2, MMP9, CRP, CST3, and CTSS expressions. Blood glucose, LDL, cholesterol, TG, creatinine, urea, and uric acid levels in the (P), (AT), and (AT + PS) groups were significantly decreased compared to the pre-experimental levels or the placebo group, while HDL, significantly increased. Furthermore, the protein and mRNA levels of NF-κB, TIMP-1, MMP2 &9, CRP, CST3, and CTSS in the (P), (AT), (AT + PS) significantly decreased compared to pre-experimental or the placebo group, while level of GLP1 and GLP1-R increased drastically. Findings suggest that purslane seed consumption alongside exercising could improve atherosclerosis plaque biomarkers through synergistically mechanisms in T2D.

Diabetes mellitus (DM) is a metabolic disease with 8% worldwide prevalence in 2011 whereby it’s prevalence estimated to reach 10% by 2030^1^. DM is characterized by hyperglycemia, glucose intolerance, abnormal lipid and protein metabolisms along with specific long–term complications affecting several major organs, including heart, blood vessels, nerves, eyes, and kidneys. Diabetic complications, such as cardiac dysfunction, atherosclerosis, neuropathy, retinopathy, and nephropathy^2^,^3^ are linked to defective insulin secretion, insulin resistance, or both^4^,^5^. Patients with type 2 diabetes (T2D) are at higher risk of complications arising from macro vascular, peripheral vascular and coronary heart disease, stroke, and insulin resistance associated with atherosclerosis^6^.

Atherosclerosis is a chronic inflammatory disease of blood vessels and it is characterized by formation of atherosclerotic plaques in arteries including calcified regions, necrotic cores, inflamed smooth muscle cells, accumulated modified lipids, endothelial cells, leukocytes, and foam cells^7^. Atherosclerosis causes cardiovascular diseases (CVD) in the components of the vascular, immune systems are involved^8^. Atherosclerosis diagnosis comprises of the identification of biomarkers linked to the development of this disease. Among the markers, nuclear factors kappa beta (NF-κB) and C-reactive protein (CRP) are the master key and prototypic markers respectively^9^,^10^. Activation of NF-κB is a key event in the pathobiology of diabetes and leads to apoptosis of beta cells. It is required for the transcription of inflammatory molecules containing adhesion molecule, cytokine and chemokines^11^. Matrix metalloproteinases (MMP) and cysteine proteases degrade extracellular matrix (ECM) proteins which are essential for vascular remodeling thus contributing to CVD^12^,^13^,^14^. High levels of glucose, LDL, VLDL, and uric acid activate CRP and NF-κB pathways which increase the expression of MMP2, MMP9 and CTSS (Fig. 1)^15^,^16^,^17^,^18^. In addition, glucagon like peptide −1 (GLP-1) is an insulinotropic agent which improves beta cell functions^19^ and protects heart muscle cells against adverse cardiac remodeling^20^.

Maintaining a healthy life style with regular physical activity has shown to reduce complications resulting from T2D^21^. In recent years, attention has been given to alternative medicines such as herbal remedies for treating and preventing plenty different diseases such as T2D^22^,^23^. *Portulaca oleracea* commonly known as purslane, is an herb from the Portulacaceae family with anti-diabetic properties^5^ and has been used for therapeutic purposes. It is listed by in the World Health Organization as one of the most used medicinal plants termed as ‘Global Panacea’^24^. *P. oleracea* is an excellent source of antioxidants such as vitamins A, C, E and β-carotene^25^,^26^. Several studies have indicated purslane extract as a factor in lowering blood sugar, triglycerides, total cholesterol, high-density-lipoprotein (HDL), low-density-lipoprotein (LDL) and body weight in diabetic rat and mice^27^,^28^. Moreover, consumption of purslane seeds alongside 8 weeks of resistance training by women with T2D, improved indicators associated with liver damage^29^, proxidant and antioxidant balance^30^ and blood pressure^31^. It also prevented exercise-induced oxidative stress^30^.

Physical activities such as resistance training promote an increase in lean muscle mass, muscle strength, the basal metabolic rate and sensitivity to insulin in diabetic individuals^32^. Regular exercising affects the regulation of enzymatic antioxidants such as catalase, super oxide dismutase and non-enzymatic antioxidants including vitamin E and C. Regular exercise is linked to CRP reduction, lipid profile regulation, increase of nitric oxide synthase, improvement in insulin sensitivity, and preservation of beta cell mass. Thus regular exercise leads to the adaptation in antioxidant capacity, protecting cells against harmful effects of oxidative stress^33^.

In this study our aim was to investigate the responses of atherosclerosis plaque biomarkers to purslane seed consumption (as non-medical intervention) along with aerobic training in women with T2D within a 16 week time period. We evaluated the lipid profile as well as traditional biomarkers and other biomarkers related with tissue inflammation and inflammatory response in diabetes patients including MMP 2&9, CRP, cystain C, creatinine, uric acid.

## Patients and Methods

### Subject recruitment

The study involved 196 women with type 2 diabetes whose fasting blood glucose levels were greater than 200 mg/dL (not taking metformin) and were diagnosed with the illness for an average of 8 years. The patients were on metformin with the dose of 500 mg/day and were registered with Tehran Hospital (Iran). Characteristics and demographics of the patients are presented in Table 1. The average age and BMI of these women were 52.08 ± 3.45 years and 29.5 ± 6.5 kg/m^2^ respectively. Eligible participants lacked any form of complications arising from acute and chronic diabetes and did not exercise regularly. The participants did not have any history of other diseases such as chronic cardiovascular and inflammatory diseases, diabetic ulcers, and hepatitis. Patients consuming vitamins and supplements or those who smoked were excluded from the study. Patients were well informed about the study prior to the experiment and written consent was obtained from them. A double-blind study method was applied and the participants were randomly assigned into 4 groups of 8; (1) placebo (PL), (2) aerobic training + placebo (AT + PL), (3) purslane seeds (PS), and (4) aerobic training + purslane seeds (AT + PS). All procedures involving experiments were carried out in strict accordance of the United States Institute of Research guidelines and approved by the Medical Centre Board of Tehran Hospital with Medical Ethics Number 4382.30.

### Plant sample preparation

Purslane seeds were purchased from a grocery shop in Tehran (Iran). The seeds were washed and air dried at room temperature for seven days. The dry seeds were powdered and packaged in capsules (5 g each). *Portulaca oleracea* was identified and verified by a botanist and deposited at Herbarium of Department of Biochemistry, (voucher specimen no. 15–04979). The consumer groups (AT + PS, PS) consumed 2.5 g of purslane seeds with lunch and 5 g with dinner daily for 16 weeks. The placebo group similarly received placebo pills (the flavored maltodextrin).

### Sample preparation and identification of compounds by Gas Chromatography/Mass Spectroscopy (GC/MS/MS)

Dried powdered (100 g) purslane seed was added with 100 ml of methanol and left at room temperature overnight. The eluate was filtered through Whatman filter paper and shade dried to remove the solvent. The extract was then weighed (0.563 g) and kept in −20 °C for further analysis. The extract was used in Gas Chromatography. The detail of Chromatography is available in supplementary file.

### Aerobic training Guideline

Patients in groups AT + PL and AT + PS performed their training under a trainer’s supervision for 16 weeks in a gym. The trainer is blinded to the subject’s treatment group. Jogging was considered for this study as a moderate-intensity aerobic activity. Progressive training was performed for a minimum of 60 min per session for three days a week at 50 to 70% of maximum heart rate (MHR) during the 16 weeks of experiment. Written informed consent was obtained from all patients before any study-related procedures were performed. Patients were familiarized with the protocol and a heart rate strap was applied on them to monitor their heart rate within the first two sessions. The training included a 15 min warm-up through walking with light static and dynamic stretching and cooling down with stretching in standing and lying position in the final 15 min. All movements were performed with medium tempo and separate movement for arms, legs and trunk. The main training time increased 40 to 45 min and heart rate has reached 70% MHR. At the end of 16 weeks participants were able to perform muscle movements with greater coordination.

### Blood Sample Collection

Blood samples were collected at the certain time of the trainings through the elbow antecubital vein of all patients, 24 hours before and after the 16 weeks training and purslane consumption for measurement of blood glucose, LDL, HDL, cholesterol, TG, creatinine, urea, uric acid, NF-κB, GLP1, GLP1R, TIMP-1, MMP2, MMP9, CRP, CST3, and CTSS mRNA and protein expressions.

### Measurement of Serum parameters

Blood samples were collected in serum separating tubes (SST) and allowed to clot at room temperature for 30 min. The blood clot was then centrifuge at 3000 g for 15 min. Aliquots of the serum samples were stored at −20 °C for further use. Fasting serum glucose and total cholesterol levels were determined using the glucose oxidase method^34^ with a digital spectrophotometer (Spectronic, US). LDL level was calculated using the Friedewald equation^35^, and to measure HDL level, diagnosis kits were used following the photometric method. Serum triglycerides was measured by using the Fossati and Prencipe method^36^. Based on manufacturer’s instructions of Fortress Diagnostics Limited, creatinine, urea, and uric acid were examined by alkaline picrate method, urease-hypochlorite and uricase-peroxidase methods respectively.

Enzyme-linked immunosorbent assay (ELISA) was performed by using commercial kits (CUSABIO - USA) for NF-κB, GLP-1, GLP1R, TIMP-1, MMP2, MMP9, CRP, CST3, and CTSS. The ELISA kits detail information are available in supplementary file.

### RNA purification and mRNA Expression Analysis by Real Time PCR (qPCR)

QIAamp RNA Blood Mini Kit (Qiagen, Germany) was used to isolate total cellular RNA from fresh whole blood. The concentration and purification of isolated RNA were evaluated by 260/280 UV absorption ratios (Gene Quant 1300, UK). Specific amplification fragments of DNA/RNA, Two-step Real time qPCR (quantitative Polymerase Chain Reaction) technique was used to calculate gene expression during the PCR amplification process with application of TaqMan reagent. This method was able to detect small differences between samples compared to other methods^37^. All reagents including probes and primers were obtained from Applied Biosystems, USA. TaqMan probe (known as fluorogenic 5′ nuclease) was chosen to perform qPCR. This probe has a sensitivity of 100% and a specificity of 96.67%^17^ and is capable of detecting as few as 50 copies of RNA/ml and as low as 5–10 molecules^18^. The same company designed specific primers for specific targets. The primers detail information are available in supplementary file.

All experiments were conducted in 3 biological replicates in a Step One Plus real time PCR machine (Applied Biosystems, USA). The Real time PCR program includes reverse transcription, at 48 °C for 15 min, activation of ampli Taq gold DNA polymerase at 95 °C for 10 min, denaturation at 95 °C for 15 sec and annealing at 60 °C for 1 min. Denaturation and annealing steps were performed for 40 cycles. The fold changes of each target per average of ACTB were calculated and considered as mRNA expression levels of the target gene. Data was analyzed according to Comparative Ct (2^−ΔΔCt^) method, where amplification of the target and the reference genes were measured in the sample and reference.

### Statistical Analysis

Levene’s equality of variable assumption was applied and the results revealed no significant differences among the observations. All data were presented as mean ± SEM. Hormone levels of each subject were analyzed by descriptive statistics. Two-way analysis of variance with repeated measure (ANOVA) was used to compare changes between groups. A Bonferroni post-hoc test was used to check for significant differences between training alone vs. purslane seeds alone vs. aerobic + purslane seeds combined. For variables with normal distribution, Pearson correlation coefficient and for non-normal distributed variable, Spearman correlation coefficient were applied. SPSS 18.0 was used in this study and p < 0.05 was considered as statistically significant.

## Results

### Gas Chromatography/Mass Spectroscopy of Purslane Seed Extract

**Figure 2:**
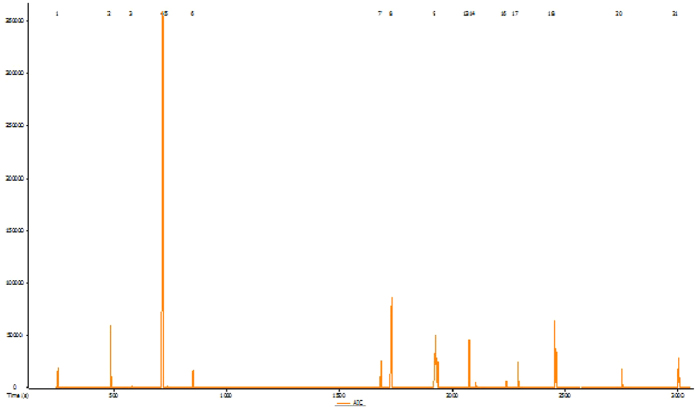
Chromatographic profile of purslane seed methanolic extract, the 21 known and unknown peaks components are identified.

The components of Purslane seed were identified and quantified by GC–MS. A total of 21 known compounds are presented in Table 2. Among these compounds, 27% are unsaturated fatty acid (linoleic and palmitoleic acid); 11.18% are phytosterols (stiotestrol), and 7.9% are saturated fatty acids (palmitic acid). Additionally, smaller amounts of unknown substances were also detected (Fig. 2).

### Serum Biochemical Parameters Analysis

The effects of purslane seed consumption and aerobic on the serum biochemicals of diabetic women are presented in Table 3. Blood glucose, LDL, Cholesterol, and TG concentration in the (PS), (AT + PL), (AT + PS) groups were significantly decreased after 16 weeks as compared to pre-experimental levels or the (PL) (p < 0.05). No significant differences observed between (PS) and (AT + PL) (p > 0.05), whereas significant interactions were detected between (PS) and (AT + PL) compared to (AT + PS) (p < 0.05).

However, the HDL levels significantly increased in all (PS), (AT + PL), (AT + PS) groups as compared to pre-experimental levels or the (PL) (p < 0.05). No significant differences observed between (PS) and (AT + PL) (p > 0.05), whereas significant distinction were detected in (PS) and (AT + PL) compared to (AT + PS) (p < 0.05). The changes were more pronounced in (AT + PS) group.

Furthermore, Creatinine, Urea, and Uric Acid in the (PS), (AT + PL), (AT + PS) groups were significantly decreased after 16 weeks as compared to the pre experimental levels or the (PL) (p < 0.05). No significant differences observed between (PS) and (AT + PL) (p > 0.05), while significant distinction were detected in (PS) and (AT + PL) compared to (AT + PS) (p < 0.05). The reductions in levels were more pronounced in (AT + PS) group.

### Atherosclerosis Biomarkers

**Figure 3:**
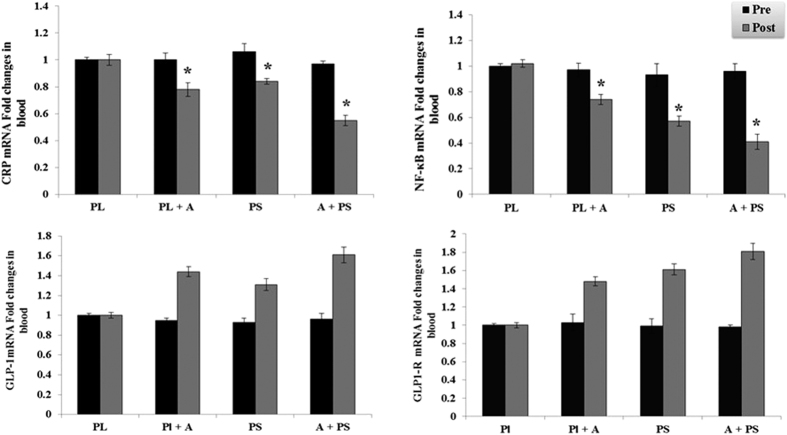
Blood CRP, NF-κB, GPL-1, and GPL1-R, mRNA expression and activity at pre or post treatment in diabetic type 2 women. Data were expressed as mean ± SEM. PL; Placebo, PL + A; Placebo + Aerobic, PS; Purslane Seed, and A + PS; Aerobic + Purslane Seed. *p < 0.05 as compared to Placebo group.

**Figure 4:**
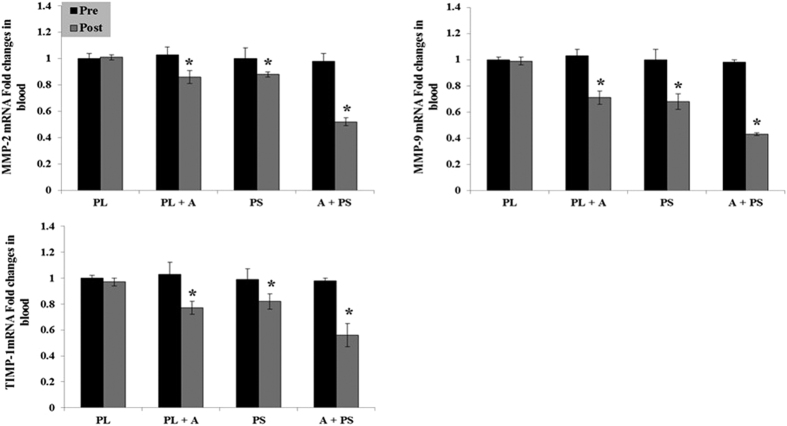
Blood MMP-2, MMP-9, and TMP-1 mRNA expression and activity at pre or post treatment in diabetic type 2 women. Data were expressed as mean ± SEM. PL; Placebo, PL + A; Placebo + Aerobic, PS; Purslane Seed, and A + PS; Aerobic + Purslane Seed. *p < 0.05 as compared to Placebo group.

**Figure 5:**
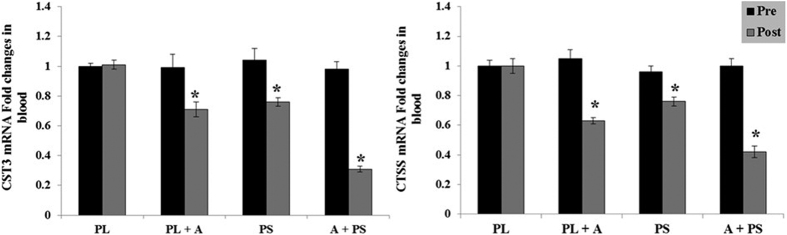
Blood CST3 and CTSS mRNA expression and activity at pre or post treatment in diabetic type 2 women. Data were expressed as mean ± SEM. PL; Placebo, PL + A; Placebo + Aerobic, PS; Purslane Seed, and A + PS; Aerobic + Purslane Seed. *p < 0.05 as compared to Placebo group.

The effects of purslane seed consumption and aerobic on the serum protein and mRNA biomarkers changes in diabetic women are presented in Table 4 and Figs 3, 4 and 5. The protein and mRNA concentration levels of NF-κB, TIMP-1, MMP 2 & 9, CRP, CST3, and CTSS in the (PS), (AT + PL), (AT + PS) had significantly decreased after 16 weeks as compared to the pre experimental levels or the (PL) (p < 0.05). No significant differences observed between (PS) and (AT + PL) in the protein and mRNA concentration levels of NF-κB, MMP2 &9, CRP, CST3, and CTSS (p > 0.05), while significant distinction were detected in (PS) and (AT + PL) compared to (AT + PS) (p < 0.05). In addition, a significant difference observed between (PS) and (AT + PL) in the protein and mRNA concentration levels of TIMP-1, as well as significant distinction in (PS) and (AT + PL) compared to (AT + PS) (p < 0.05).

Furthermore, GLP1 and GLP1-R had profoundly increased in the (PS), (AT + PL), (AT + PS) as compared to the pre experimental levels or the (PL) (p < 0.05). No significant difference observed between (PS) and (AT + PL) in the protein and mRNA concentration levels of GLP1-R (p > 0.05), while a significant difference observed between (PS) and (AT + PL) in the protein and mRNA concentration levels of GLP1 (p < 0.05). Moreover, significant distinction were detected in the protein and mRNA concentration levels of GLP1 and GLP1-R in (PS) and (AT + PL) compared to (AT + PS) (p < 0.05). The changes were more pronounced in (AT + PS) group.

## Discussion

The control and treatment of diabetes and its complications predominantly depends on chemical or biochemical agents. However, total recovery from diabetes has never been reported^38^,^39^. The data from the current study showed that 16 weeks of aerobic training or purslane seed consumption ere effective in reduction of markers of inflammations such as NF-κB, CRP, CST3, CTSS, MMP 2 & 9 and TIMP-1 in diabetic patients. In addition, glucose, TG, LDL, urea, uric acid and creatinine levels in all treated groups were significantly reduced. Furthermore, there was an increase in the levels of HDL, GLP-1 and GLP-1R. The improvement was more remarkable in women who received training and supplementation simultaneously. The changes in the level of biomarkers were further evaluated by the expression of mRNA from blood samples of the same subjects. Our results show that the mRNA levels of NF-κB, CRP, CST3, CTSS, MMP 2, 9 and TIMP-1 increased after intervention. GC-MS analyses results indicated the presence of 21 compounds, of which 27% are poly unsaturated fatty acid (linoleic and palmetoleic acid) and 11.18% are phytosterols (stiotestrol). The observed effects of purslane seeds may be due to the presence of these compounds.

The findings suggest that glucose levels had decreased in all groups. However the decrease was more evident in the group consuming purslane seed and exercising simultaneously. Purslane seed consumption has been shown previously to impact glucose levels^27^. High levels of glucose in diabetes appears to be an initiating factor for the eventual cascade of biomarkers involved in inflammation and coronary heart diseases. Improvements in lipid profile were observed in the subjects after 16 weeks of aerobic training or purslane seed consumption or both (Table 3). The reduction of lipid profile in the aerobic group could be due to the decrease of free fatty acids, which negatively affect insulin resistance and excess lipid availability^40^,^41^,^42^,^43^,^44^,^45^. Hence, aerobic training is beneficial for patients with T2D^40^,^42^,^46^. The results obtained from our study also highlights the positive effects of purslane seed consumption on TG, LDL, cholesterol and HDL levels, which is consistent with earlier reports in T2D of Yemeni and Iranian patients^5^,^31^.

Levels of blood urea, uric acid and creatinine (Table 3) were reduced in all treated groups. In T2D overweight patients, uric acid and urea serve as biomarkers for impaired physical performances and are associated with blood glucose levels^47^. Purslane consumption in T2D mice^48^ and T2D women has also been shown to reduce levels of creatinine, uric acid and urea^49^. Therefore, the reduction of blood nitrogen content seen in this study, may be lined to reduction in glucose levels (Table 3) following purslane seed consumption or aerobic training. In a study reported by Sousa^50^, twelve weeks of resistance training improved uric acid levels in T2D in Brazilian patients^50^,^51^,^52^,^53^. However, six months of aerobic training did not produce remarkable effects on creatinine and urea in untrained aged healthy women^54^.

The current study highlighted the beneficial effects of purslane seed consumption in alleviation of diabetes parameters. The beneficial effect may due to the presence of unsaturated fatty acids and beta-sistosterol as identified via GC-MS analysis (Table 2 and Fig. 2). Previous reports have suggested that, unsaturated fatty acids are responsible in reducing levels of LDL and cholesterol synthesis^55^, enhancing insulin function^56^ and improving glucose tolerance and lipid profile^57^,^58^. Another active substance in purslane seed is beta-sitosterol which is a phytosterol. Studies have found that beta-sitosterol has cholesterol^59^ and LDL^60^ lowering effects, increases the expression of VEGF (vascular endothelial growth factor) and FLK-1 (Fetal Liver Kinase 1) of VEGF receptors while modulating inflammation and regulation the immune systems. The effectiveness of purslane seed consumption is in lowering cholesterol levels explained by the synergistic effect of both phytosterols and unsaturated fatty acids^61^.

Hyperglycemia, hyperlipidemia, hyperinsulinemia^17^,^18^,^53^ and hyperuricemia^62^,^63^,^64^ of diabetics are common symptoms which enhance production of pro inflammatory markers such as CRP, IL6, TNFα, NF-κB^17^,^18^,^53^,^62^,^64^, reactive oxygen species (ROS) and reduce anti-inflammatory cytokine and adiponectin, which are involved in insulin resistance^16^,^65^ and diabetes^66^. Reduction in the level of NF-κB and CRP in the inflammatory cascade was observed in treated groups of diabetics’ subjects. Our experiments showed that purslane seed consumption resulted in reduction of CRP and NF-κB (Table 4 and Fig. 3). This may be the result of inhibitory effects of unsaturated fatty acids on NF-κB reported in diabetic patients^67^. The inhibitory effect of unsaturated fatty acids such as omega 3 on CRP^68^ in diabetic patients was reported even though the changes in CRP level were not significant. However, the significant reduction of CRP in the present study may indicate synergistic effect of various compounds found in purslane seed. Reduction in the level of CRP following resistance or strength training has been reported in patients with kidney disease^45^,^69^, older adults with T2D^21^ and diabetic men^70^ and in improving insulin sensitivity^21^. Various types of exercise may affect levels of NFκB differently. For instance, acute exercise did not change the level of NFκB in T2D patients^53^, but acute treadmill running raised the level of NFκB activity in rat skeletal muscles^71^. Therefore, reduction in levels of NFκB may be linked to CRP level reduction following aerobic exercise (Table 4) by impact on different synergistically pathways^52^,^53^,^72^. The expression of NFκB and CRP are interconnected whereby CRP enhances NFκB levels^9^, thus aerobic training and/or purslane seed consumption may directly or indirectly reduce the levels of NFκB via reducing the CRP.

The reduction of these two biomarkers is associated with the levels of inflammation and ROS in addition to regulation of ECM^9^,^18^. Matrix metalloproteinase (MMPs) and cathepsin S (CTSS) are proteases that degrade at least one component ECM and contribute for tissue remodeling and inflammation. Higher level of CRP and IL-6 are associated with higher level of CTSS^17^ and higher levels of NFκB enhance MMPs level^16^,^73^. In pathological conditions such as diabetes, the balance between the protease enzymes (MMPs and CTSS) and their inhibitors (metallopeptidase inhibitor 1; TIMP-1 and CST3) are dysregulated^74^. The concentrations of MMP-2, 9 and TIMP-1 has been shown higher in T2D patients as compared to non-diabetic subjects^75^,^76^ but in the current study, their levels were significantly reduced in all treated groups following different interventions (Table 4 and Fig. 4). Positive effects of exercise improve the balance between pro- and anti-inflammatory and oxidative stress markers which could improve nitric oxide bioavailability and alter MMPs and/or tissue inhibitors of MMPs (TIMP) activity. Chronic exercise training, that acts as a mechanical stressor for the arteries, could exert its effects by altering MMP/TIMPs activity directly and/or indirectly via the anti-inflammatory and oxidative stress responses of exercise^77^. The finding were concurrent with those of Kim *et al*.^18^ which states that the level of MMP-2 was reduced after low intensity exercise training in diabetic mice^78^. The suppressive effect of purslane seed on MMP and TIMP activity may be caused by the presence of the unsaturated fatty acids in the seeds. Similar outcomes were observed in patients with multiple sclerosis^79^ and in pregnant rats^80^.

CTSS is another important regulator of inflammation^14^ that plays a role in pathological conditions such as diabetes^17^,^81^. This regulator and its inhibitor CST3 are higher in diabetic patients as compared to healthy subjects^82^. Nonetheless the current interventions applied in this study reduced the protein and mRNA of both CTSS and CST3 (Table 4 and Fig. 5). This reduction following exercise may changes energy balance in adipose tissues^83^. Moreover, CTSS is positively correlated with weight loss^83^,^84^ and TG level in obese subjects^85^. Reduction in CTSS and LDL was also observed in diet induced weight loss in non-obese men and women^86^. This further supports the hypothesis that CTSS contributes to cardiovascular risks in obesity^83^. The reduction of CTSS and CST3 was also observed in the group consuming purslane seed alone which could be due to the presence of unsaturated fatty acids and beta-sistosterol found in the seeds. However, the mechanism of for their reduction is unknown.

This study also evaluated the effect of intervention on biomarkers for insulin and beta cell activity. The levels of GLP-1 and GLP-1R were measured. GLP-1 is an insulinotropic in T2D which improves insulin secretion via it’s receptor and by stimulation of glucose dependent insulin secretion^19^. It also has protective effect against degradation of ECM^20^ and inhibits apoptosis of beta cells^87^. In addition GLP-1 mimetics are potential drugs to treat T2D^88^. The present findings, showed improving effects of interventions on GLP-1 and its receptor levels (Table 4 and Fig. 3). This is possibly linked to effects of purslane and aerobic training on beta cells mediated by the AMPK activated pathway^88^,^89^. Exercise and physical activity induce the AMPK pathway and inhibit energy consuming pathways such as fatty acid and cholesterol synthesis and stimulate the ATP catabolic pathway^90^. Since AMPK signaling pathway is among the crucial pathways involved in inflammation, following activation of this pathway, inhibition of NFκB and its downstream signaling pathway occurs^91^.

Thus the reduction in the inflammatory state of diabetes may be an important factor leading to improved insulin sensitivity and better metabolic control^21^. Increased physical activity and weight loss are associated with lower CRP and reduction in concentration of other markers of inflammation^33^,^92^. Therefore, both intervention alone or in combination might act through AMPK pathway and inhibit the activation of downstream inflammation through inhibition of CRP and NFκB. Among different insulin signaling pathways, available treatments for diabetes usually trigger the AMPK pathway which is insulin independent^93^ and metformin activated that results in the inhibition of NF-kB activity in endothelial cells^94^,^95^. In this study, the level of NF-kB as a master inflammation key was reduced with purslane intake and/or aerobic training which could be due to activation of the AMPK pathway. Previous reports have indicated that aerobic exercise^52^,^53^ and the uptake of unsaturated fatty acids activate the AMP and AMPK pathways^96^.

## Conclusion

The data obtained from this study indicated that 16 weeks of aerobic training or/and purslane seed consumption ere effective in regulation of diabetic parameters and biomarkers associated with atherosclerosis in women with T2D. This may be due to the synergistic effect of aerobic training and unsaturated fatty acids found in purslane seed which activate the AMP and AMPK pathways. This may result in the regulation of biomarkers involved in cardiovascular complications of T2D. The parallel effects of purslane intake and aerobic training in their simultaneous role were more prominent which could be a strong therapeutic effective factor on diabetic patients with CVD. Thus, with the benefits of this combination on reducing health risk factors, diabetic patients are advised to exploit this arrangement of alternative to control and manage their disease. However, further investigations are warranted, and such research should focus on identifying the specific properties of purslane seed, proper dose, role of various active compounds, as well as the possibilities for synergistic interactions not only between the various components of this seed, but also between various alternatives in treating and reducing symptoms of these diseases.

## Additional Information

**How to cite this article**: Dehghan, F. *et al*. Purslane (Portulaca oleracea) Seed Consumption And Aerobic Training Improves Biomarkers Associated with Atherosclerosis in Women with Type 2 Diabetes (T2D). *Sci. Rep.* **6**, 37819; doi: 10.1038/srep37819 (2016).

**Publisher's note:** Springer Nature remains neutral with regard to jurisdictional claims in published maps and institutional affiliations.

## Supplementary Material

Supplementary Information

## Figures and Tables

**Figure 1:**
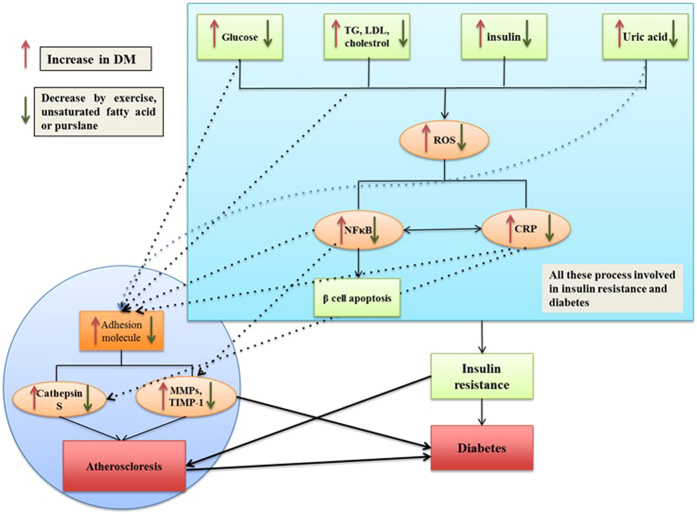
Schematic illustration of some of the key pathological pathways that are involved in both type 2 diabetes and atherosclerosis. Hyperglycemia, hyperlipidemia, hyperinsulinia, and hyperuricimia increase ROS generation through oxidative stress. ROS activates a number of stress sensitive kinase which mediates insulin resistance via activation of NF-κB and CRP. On the other hand, activation of NF-κB and CRP affect the structure of extracellular matrix and increase the activity of MMP and cathepsin S which are involved in atherosclerosis and diabetes. Exercise and purslane intake improve the regulation of biomarkers involved in the activation of these pathways. TG; triglycerides, LDL; Low-density lipoprotein, ROS; reactive oxygen species, NF-κB; nuclear factor kappa-light-chain-enhancer of activated B cells, CRP; C-reactive protein, MMPs; Matrix metalloproteinase, TIMP-1; metallopeptidase inhibitor 1.

**Table 1:** Characteristics and demographics of the diabetics’ subjects.

	Placebo (PL) Mean ± SEM	Aerobic Training + Placebo (AT + PL) Mean ± SEM	Purslane Seeds (PS) Mean ± SEM	Aerobic Training + Purslane Seeds (AT + PS) Mean ± SEM
Age, year	50.17 ± 5.34	58.83 ± 6.79	52.33 ± 4.08	61.17 ± 4.88
Height, cm	160.67 ± 6.44	162.50 ± 6.53	159.17 ± 6.65	154.50 ± 10.60
Weight, cm	75.67 ± 9.44	79.50 ± 8.96	73.50 ± 6.65	70.83 ± 7.88
BMI, Kg/m^2^	29.8 ± 6.4	29.5 ± 7.2	29.0 ± 5.0	29.9 ± 7.3

**Table 2:** Composition of total components contents of purslane seeds.

Peak #	Name	R.T. (s)	Area	Relative Amount (RA)	Mass
1	2-Propanol, 1-[(1-methylethyl) amino]-3-[2-(2-propenyl) phenoxy]-(Alprenolol, or alfeprol, alpheprol)	252.15	311449	0.04	249
2	4-Methylpiperidine-1-carboxylic acid, phenyl ester (acid amin3)	485.35	6340394	0.81	219
3	N-(1-Methoxycarbonyl-1-methylethyl)-4-methyl-2-aza-1,3-dioxane	582.25	4477291	0.57	203
4	Tetrahydropyran-4-ol, 2,2-dimethyl-4-[[(thiophen-2-ylmethyl) amino]methyl]-	717.9	129487357	16.61	255
5	1,3-Oxathiolane, 2-[[(2-chloroethyl) thio]methyl]-2-methyl-	736.3	388091	0.05	212
6	Benzoic acid, 2-(2-isopropyl-5-methylphenoxymethyl)-	853	6451312	0.83	284
7	29.82 Methyl dodecanoate	1684.55	2410439	0.31	214
8	n-Hexadecanoic acid (Palmitic acid)	1731.6	61905629	7.94	256
9	Decanamide, N-(2-hydroxyethyl)-	1925.3	52686347	6.76	215
10	9,12-Octadecadienoic acid (Z,Z)- Linoleic acid (LA)	1927.4	120792530	15.49	280
11	9-Hexadecenoic acid-(Palmitoleic acid)	1933.25	93736575	12.02	254
12	9,12,15-Octadecatrien-1-ol, (Z,Z,Z)-	1934.05	24934192	3.20	264
13	S-[2-[N,N-Dimethylamino]ethyl]N,N-dimethylcarbamoyl thiocarbohydroximate	2074.5	4196678	0.54	219
14	Palmidrol	2103.5	10489224	1.35	299
15	4-(7-Methoxy-7-methyloxepan-2-ylidene) butan-2-one	2110.3	5920620	0.76	212
16	6-Methoxy-2-methyl-quinoline-3-carboxylic acid 2-dimethylamino-ethyl ester	2240.95	16583058	2.13	288
17	Glycerol 1-palmitate	2292.1	24680340	3.17	330
18	Z-5,17-Octadecadien-1-ol acetate	2453.7	79862448	10.24	308
19	9,12,15-Octadecatrien-1-ol, (Z,Z,Z)-	2460.85	29437987	3.78	264
20	3H-Imidazo[4,5-b]pyridine, 2-(2-ethylhexylsulfanyl)-	2753	17494020	2.24	263
21	beta-Sitosterol	3004.8	87157789	11.18	414
	**Total**		**779,743,770**	**100**	

**Table 3:** Changes in the serum variables of diabetic subjects between 4 groups pre and post 16 weeks experiments.

Variable	Stage	Placebo (PL) Mean ± SEM	Aerobic Training + Placebo (AT + PL) Mean ± SEM	Purslane Seeds (PS) Mean ± SEM	Aerobic Training + Purslane Seeds (AT + PS) Mean ± SEM
Glucose, mg/dL	Pre Post	168.2 ± 18.1	164.8 ± 17.5	167.4 ± 14.1	167.6 ± 20.6
		173.1 ± 19.7	147.3 ± 18.3*^,†^	152.1 ± 11.9*^,^†	140.1 ± 15.2*^,^†^,^#^,^Ω
LDL-C, mg/dL	Pre Post	83.3 ± 8.6	87.1 ± 12.4	84.1 ± 14.2	86.3 ± 11.7
		82.1 ± 19.7	75.5 ± 17.2*^,^†	79.9 ± 12.6*^,^†	72.8 ± 19.7*^,^†^,^#^,^Ω
HDL-C, mg/dL	Pre Post	35.8 ± 4.9	36.7 ± 8.3	35.3 ± 7.2	34.9 ± 3.7
		33.6 ± 9.1	39.8 ± 10.3*^,^†	39.2 ± 6.9*^,^†	46.7 ± 7.4*^,^†^,^#^,^Ω
Cholesterol, mg/dL	Pre Post	197.2 ± 22.4	198.5 ± 26.1	196.9 ± 22.6	198.7 ± 24.8
		209.2 ± 20.1	169.7 ± 14.8*^,^†	172.3 ± 19.3*^,^†	155.2 ± 16.4*^,^†^,^#^,^Ω
TG, mg/dL	Pre Post	183.1 ± 26.3	182.8 ± 21.7	183.6 ± 27.2	183.1 ± 23.8
		182.9 ± 24.7	152.7 ± 29.2*^,^†	159.3 ± 22.0*^,^†	142.2 ± 21.5*^,^†^,^#^,^Ω
Creatinine, mg/dL	Pre Post	1.43 ± 0.04	1.41 ± 0.05	1.40 ± 0.05	1.41 ± 0.03
		1.43 ± 0.02	0.81 ± 0.06*^,^†	0.88 ± 0.06*^,^†	0.42 ± 0.07*^,^†^,^#^,^Ω
Urea, mg/dL	Pre Post	19.31 ± 1.19	18.99 ± 1.04	18.86 ± 0.82	19.01 ± 0.63
		19.31 ± 1.11	14.21 ± 0.98*^,^†	14.42 ± 0.07*^,^†	10.62 ± 0.98*^,^†^,^#^,^Ω
Uric Acid mg/dL	Pre Post	4.65 ± 0.15	4.82 ± 0.18	4.59 ± 0.16	4.71 ± 0.15
		4.66 ± 0.42	3.38 ± 0.21*^,^†	3.11 ± 0.14*^,^†	2.39 ± 0.23*^,^†^,^#^,^Ω

^*^Denote significant differences from pre-test.

^†^Denote significant differences from Placebo group.

^#^Denote significant differences from Aerobic Training+ Placebo.

^Ω^Denote significant differences from Purslane Seeds group.

p < 0.05 as compared to pre-test, Placebo, Aerobic Training+ Placebo, and Purslane Seeds groups.

**Table 4:** Changes in the Serum biomarkers concentration diabetic subjects between 4 groups pre and post 16 weeks experiments.

Variable	Stage	Placebo (PL) Mean ± SEM	Aerobic Training+ Placebo (AT + PL) Mean ± SEM	Purslane Seeds (PS) Mean ± SEM	Aerobic Training+Purslane Seeds (AT + PS) Mean ± SEM
NF-κB1, ng/mL	Pre Post	8.74 ± 0.7	8.87 ± 0.5	8.52 ± 0.5	8.79 ± 0.6
		8.69 ± 0.2	6.7 ± 0.3$^,^β	6.5 ± 0.4$^,^β	5.3 ± 0.4$^,^β^,^€^,^£
GLP1, ng/mL	Pre Post	5.44 ± 0.3	4.38 ± 0.6	3.82 ± 0.3	3.43 ± 0.3
		5.55 ± 0.4	5.78 ± 0.3$^,^β	4.93 ± 0.6$^,^β^,^€	5.77 ± 0.7$^,^β^,^€^,^£
GLP1-R, ng/mL	Pre Post	0.56 ± 0.012	0.52 ± 0.016	0.48 ± 0.017	0.49 ± 0.011
		0.59 ± 0.018	0.69 ± 0.013$^,^β	0.67 ± 0.015$^,^β	0.71 ± 0.014$^,^β^,^€^,^£
TIMP-1, ng/mL	Pre Post	10.72 ± 0.56	10.47 ± 0.47	10.22 ± 0.68	10.86 ± 0.87
		10.69 ± 0.6	6.98 ± 0.95$^,†,^β	7.84 ± 0.44$^,^β^,^€	5.12 ± 0.65$^,^β^,^€^,^£
MMP2, ng/mL	Pre Post	0.88 ± 0.02	0.87 ± 0.03	0.88 ± 0.02	0.86 ± 0.02
		0.87 ± 0.03	0.70 ± 0.04$^,†,^β	0.67 ± 0.03$^,^β	0.55 ± 0.04$^,^β^,^€^,^£
MMP9, ng/mL	Pre Post	1.62 ± 0.05	1.59 ± 0.07	1.58 ± 0.08	1.60 ± 0.07
		1.61 ± 0.04	1.08 ± 0.22$^,†,^β	1.05 ± 0.15$^,^β	0.55 ± 0.19$^,^β^,^€^,^£
CRP, mg/mL	Pre Post	8.72 ± 0.4	7.91 ± 0.6	7.85 ± 0.6	8.56 ± 0.7
		8.83 ± 0.5	6.1 ± 0.3$^,^β	6.2 ± 0.1$^,^β	5.1 ± 0.3$^,^β^,^€^,^£
CST3, ng/mL	Pre Post	35.13 ± 4.32	35.33 ± 3.52	34.71 ± 4.61	34.51 ± 3.22
		35.75 ± 3.25	26.25 ± 3.95$^,^β	25.98 ± 2.44$^,^β	20.09 ± 3.65$^,^β^,^€^,^£
CTSS, ng/mL	Pre Post	0.77 ± 0.02	0.76 ± 0.03	0.79 ± 0.06	0.79 ± 0.03
		0.77 ± 0.02	0.64 ± 0.05$^,^β	0.61 ± 0.02$^,^β	0.29 ± 0.02$^,^β^,^€^,^£

^$^Denote significant differences from pre-test.

^β^Denote significant differences from Placebo group.

^€^Denote significant differences from Aerobic Training+ Placebo.

^£^Denote significant differences from Purslane Seeds group.

p < 0.05 as compared to pre-test, Placebo, Aerobic Training+ Placebo, and Purslane Seeds groups.

## References

[b1] Shamima A., Rahman M., Krull S. A. & Sultana P. Prevalence of diabetes and prediabetes and their risk factors among Bangladeshi adults: a nationwide survey. Bulletin of the World Health Organization 92, 204–213A (2014). 24700980 10.2471/BLT.13.128371 PMC3949596

[b2] Fadini G. P., Iori E., Marescotti M. C., de Kreutzenberg S. V. & Avogaro A. Insulin-induced glucose control improves HDL cholesterol levels but not reverse cholesterol transport in type 2 diabetic patients. Atherosclerosis 235, 415–417 (2014). 24933032 10.1016/j.atherosclerosis.2014.05.942

[b3] Patiño M. N. . Caries, periodontal disease and tooth loss in patients with diabetes mellitus types 1 and 2. Acta odontologica latinoamericana: AOL 21, 127–133 (2007). 19177848

[b4] Perez-Gallardo R. V. . Effects of diabetes on oxidative and nitrosative stress in kidney mitochondria from aged rats. J Bioenerg Biomembr 46, 511–518 (2014). 25425473 10.1007/s10863-014-9594-4

[b5] El-Sayed M. I. Effects of Portulaca oleracea L. seeds in treatment of type-2 diabetes mellitus patients as adjunctive and alternative therapy. J Ethnopharmacol 137, 643–651 (2011). 21718775 10.1016/j.jep.2011.06.020

[b6] Laakso M. Cardiovascular disease in type 2 diabetes from population to man to mechanisms: the Kelly West Award Lecture 2008. Diabetes Care 33, 442–449 (2010). 20103560 10.2337/dc09-0749 PMC2809299

[b7] Galkina E. & Ley K. Immune and inflammatory mechanisms of atherosclerosis. Annual review of immunology 27, 165 (2009). 10.1146/annurev.immunol.021908.132620 PMC2734407 19302038

[b8] Watala C. & Winocour P. The relationship of chemical modification of membrane proteins and plasma lipoproteins to reduced membrane fluidity of erythrocytes from diabetic subjects. Clinical Chemistry and Laboratory Medicine 30, 513–520 (1992). 10.1515/cclm.1992.30.9.513 1457612

[b9] Chang J. W. . C-reactive protein induces NF-kappaB activation through intracellular calcium and ROS in human mesangial cells. Nephron Exp Nephrol 101, e165–172 (2005). 16131811 10.1159/000087940

[b10] Jialal I. & Devaraj S. The Role of C-Reactive Protein Activation of Nuclear Factor Kappa-B in the Pathogenesis of Unstable Angina*. Journal of the American College of Cardiology 49, 195–197 (2007). 17222730 10.1016/j.jacc.2006.10.018

[b11] Patel S. & Santani D. Role of NF-kappa B in the pathogenesis of diabetes and its associated complications. Pharmacol Rep 61, 595–603 (2009). 19815941 10.1016/s1734-1140(09)70111-2

[b12] Song W. & Ergul A. Type-2 diabetes-induced changes in vascular extracellular matrix gene expression: relation to vessel size. Cardiovasc Diabetol 5, 3 (2006). 16503991 10.1186/1475-2840-5-3 PMC1434726

[b13] Cheng X. W. . Role for Cysteine Protease Cathepsins in Heart Disease: Focus on Biology and Mechanisms With Clinical Implication. Circulation 125, 1551–1562 (2012). 22451605 10.1161/CIRCULATIONAHA.111.066712

[b14] Jobs E. . Serum cathepsin S is associated with decreased insulin sensitivity and the development of type 2 diabetes in a community-based cohort of elderly men. Diabetes Care 36, 163–165 (2013). 22923671 10.2337/dc12-0494 PMC3526243

[b15] Kowluru R. A., Zhong Q. & Santos J. M. Matrix metalloproteinases in diabetic retinopathy: potential role of MMP-9. Expert Opin Investig Drugs 21, 797–805 (2012). 10.1517/13543784.2012.681043 PMC3802521 22519597

[b16] Lorenzo O. . Potential Role of Nuclear Factor κB in Diabetic Cardiomyopathy. Mediators of Inflammation 2011 (2011). 10.1155/2011/652097 PMC3136091 21772665

[b17] Jobs E. . Serum cathepsin S is associated with serum C-reactive protein and interleukin-6 independently of obesity in elderly men. J Clin Endocrinol Metab 95, 4460–4464 (2010). 20610597 10.1210/jc.2010-0328

[b18] Green C. J., Pedersen M., Pedersen B. K. & Scheele C. Elevated NF-κB Activation Is Conserved in Human Myocytes Cultured From Obese Type 2 Diabetic Patients and Attenuated by AMP-Activated Protein Kinase. Diabetes 60, 2810–2819 (2011). 21911750 10.2337/db11-0263 PMC3198079

[b19] Kim W. & Egan J. M. The role of incretins in glucose homeostasis and diabetes treatment. Pharmacol Rev 60, 470–512 (2008). 19074620 10.1124/pr.108.000604 PMC2696340

[b20] Tate M., Robinson E., McDermott B. J. & Grieve D. J. Glucagon-like peptide-1 protects against cardiac dysfunction and extracellular matrix remodelling IN experimental diabetes. Heart 98, A1 (2012).

[b21] Brooks N. . Strength training improves muscle quality and insulin sensitivity in Hispanic older adults with type 2 diabetes. Int J Med Sci 4, 19–27 (2007). 10.7150/ijms.4.19 PMC1752232 17211497

[b22] Dehghan F. . Saffron with resistance exercise improves diabetic parameters through the GLUT4/AMPK pathway *in-vitro* and *in-vivo*. Sci Rep 6, 25139 (2016). 27122001 10.1038/srep25139 PMC4848502

[b23] Ranjbari A. . *In vivo* and *in vitro* evaluation of the effects of Urtica dioica and swimming activity on diabetic factors and pancreatic beta cells. BMC Complement Altern Med 16, 101 (2016). 26980377 10.1186/s12906-016-1064-6 PMC4791772

[b24] Shanker N. & Debnath S. Impact of dehydration of purslane on retention of bioactive molecules and antioxidant activity. J Food Sci Technol 52, 6631–6638 (2015). 26396410 10.1007/s13197-015-1741-3 PMC4573115

[b25] Liu L. . Fatty acids and beta-carotene in australian purslane (Portulaca oleracea) varieties. J Chromatogr A 893, 207–213 (2000). 11043602 10.1016/s0021-9673(00)00747-0

[b26] Uddin M. K. . Purslane weed (Portulaca oleracea): a prospective plant source of nutrition, omega-3 fatty acid, and antioxidant attributes. ScientificWorldJournal 2014, 951019 (2014). 24683365 10.1155/2014/951019 PMC3934766

[b27] Gong F. . Hypoglycemic effects of crude polysaccharide from Purslane. International Journal of Molecular Sciences 10, 880–888 (2009). 19399226 10.3390/ijms10030880 PMC2672007

[b28] Abdalla H. M. Jr. Purslane extract effects on obesity-induced diabetic rats fed a high-fat diet. Malays J Nutr 16, 419–429 (2010). 22691995

[b29] Salehi A. & Farzanegi P. Effect of 8 weeks of resistance training with and without portulacalo seeds on some of liver injury markers in women with diabetes type 2. URMIA MEDICAL JOURNAL 25, 968–978 (2015).

[b30] Fakoory Jouybari M., Farzanegi P. & A. B. The effect of 8-week aerobic exercise with purslane supplementation consumption on peroxidant and antioxidants indicators in women with type 2 diabetes. J Shahid Sadoughi Univ Med Sci 22, 928–939 (2014).

[b31] Esmaillzadeh A., Zakizadeh E., Faghihimani E., Gohari M. & Jazayeri S. The effect of purslane seeds on glycemic status and lipid profiles of persons with type 2 diabetes: A randomized controlled cross-over clinical trial. J Res Med Sci 20, 47–53 (2015). 25767522 PMC4354065

[b32] Ciolac E. G. & Physical G. G. exercise and metabolic syndrome. Rev Bras Med Esporte 10, 319–324 (2004).

[b33] de Lemos E. T., Oliveira J., Pinheiro J. P. & Reis F. Regular physical exercise as a strategy to improve antioxidant and anti-inflammatory status: benefits in type 2 diabetes mellitus. Oxid Med Cell Longev 2012, 741545 (2012). 22928086 10.1155/2012/741545 PMC3425959

[b34] Raabo B. E. & Terkildsen T. On the enzymatic determination of blood glucose. Scandinavian journal of clinical and laboratory investigation 12, 402–407 (1960). 13738785 10.3109/00365516009065404

[b35] Friedewald W. T., Levy R. I. & Fredrickson D. S. Estimation of the concentration of low-density lipoprotein cholesterol in plasma, without use of the preparative ultracentrifuge. Clinical chemistry 18, 499–502 (1972). 4337382

[b36] Fossati P. & Prencipe L. Serum triglycerides determined colorimetrically with an enzyme that produces hydrogen peroxide. Clinical chemistry 28, 2077–2080 (1982). 6812986

[b37] Wong M. L. & Medrano J. F. Real-time PCR for mRNA quantitation. Biotechniques 39, 75 (2005). 16060372 10.2144/05391RV01

[b38] Li W., Zheng H., Bukuru J. & De Kimpe N. Natural medicines used in the traditional Chinese medical system for therapy of diabetes mellitus. Journal of Ethnopharmacology 92, 1–21 (2004). 15099842 10.1016/j.jep.2003.12.031

[b39] Ivorra M., Paya M. & Villar A. A review of natural products and plants as potential antidiabetic drugs. Journal of Ethnopharmacology 27, 243–275 (1989). 2693840 10.1016/0378-8741(89)90001-9

[b40] Chien K.-L., Chen M.-F., Hsu H.-C., Su T.-C. & Lee Y.-T. Sports activity and risk of type 2 diabetes in Chinese. Diabetes Research and Clinical Practice 84, 311–318 (2009). 19359062 10.1016/j.diabres.2009.03.006

[b41] Gordon L. A. . Effect of exercise therapy on lipid profile and oxidative stress indicators in patients with type 2 diabetes. BMC Complement Altern Med 8, 21 (2008). 18477407 10.1186/1472-6882-8-21 PMC2390515

[b42] Hayashino Y., Jackson J. L., Fukumori N., Nakamura F. & Fukuhara S. Effects of supervised exercise on lipid profiles and blood pressure control in people with type 2 diabetes mellitus: A meta-analysis of randomized controlled trials. Diabetes Research and Clinical Practice 98, 349–360 (2012). 23116535 10.1016/j.diabres.2012.10.004

[b43] Miyatake N. . Daily walking reduces visceral adipose tissue areas and improves insulin resistance in Japanese obese subjects. Diabetes Res Clin Pract 58, 101–107 (2002). 12213351 10.1016/s0168-8227(02)00129-8

[b44] Ross R. . Reduction in obesity and related comorbid conditions after diet-induced weight loss or exercise-induced weight loss in men. A randomized, controlled trial. Ann Intern Med 133, 92–103 (2000). 10896648 10.7326/0003-4819-133-2-200007180-00008

[b45] Saremi A., Mosleh abadi M. & Parastesh M. ffects of Twelve-week Strength Training on Serum Chemerin, TNF-α and CRP Level in Subjects with the Metabolic Syndrome. Iranian Journal of Endocrinology and Metabolism 12, 536–543 (2011).

[b46] Davey Smith G. . Incidence of type 2 diabetes in the randomized multiple risk factor intervention trial. Ann Intern Med 142, 313–322 (2005). 15738450 10.7326/0003-4819-142-5-200503010-00006

[b47] Bo S., Cavallo-Perin P., Gentile L., Repetti E. & Pagano G. Hypouricemia and hyperuricemia in type 2 diabetes: two different phenotypes. Eur J Clin Invest 31, 318–321 (2001). 11298778 10.1046/j.1365-2362.2001.00812.x

[b48] Lee A. S. . An aqueous extract of Portulaca oleracea ameliorates diabetic nephropathy through suppression of renal fibrosis and inflammation in diabetic db/db mice. Am J Chin Med 40, 495–510 (2012). 22745066 10.1142/S0192415X12500383

[b49] Farzanegi P., Pour Amin Z. & Habibian M. Changes of Liver Trans-Aminases after a Period of Selected Aerobic Training in Postmenopausal Women. Medical Labrotoary Journal 8, 22–28 (2014).

[b50] Sousa M. S. . Resistance Training in Type 2 Diabetic Patients Improves Uric Acid levels. J Hum Kinet 43, 17–24 (2014). 25713640 10.2478/hukin-2014-0085 PMC4332177

[b51] Bodine S. C. mTOR signaling and the molecular adaptation to resistance exercise. Med Sci Sports Exerc 38, 1950–1957 (2006). 17095929 10.1249/01.mss.0000233797.24035.35

[b52] Sriwijitkamol A. . Reduced skeletal muscle inhibitor of kappaB beta content is associated with insulin resistance in subjects with type 2 diabetes: reversal by exercise training. Diabetes 55, 760–767 (2006). 16505240 10.2337/diabetes.55.03.06.db05-0677

[b53] Tantiwong P. . NF-kappaB activity in muscle from obese and type 2 diabetic subjects under basal and exercise-stimulated conditions. Am J Physiol Endocrinol Metab 299, E794–801 (2010). 20739506 10.1152/ajpendo.00776.2009 PMC2980364

[b54] Bijeh N. & Farahati S. The Effect of Six Months of Aerobic training on Renal Function Markers in Untrained Middle-Aged Women. Int J Sports Studies 3, 218–224 (2013).

[b55] Nestel P. J. Fish oil and cardiovascular disease: lipids and arterial function. The American Journal of Clinical Nutrition 71, 228S–231S (2000). 10617976 10.1093/ajcn/71.1.228S

[b56] Oh J. Y. Serum cystatin C as a biomarker for predicting coronary artery disease in diabetes. Korean Diabetes J 34, 84–85 (2010). 20548839 10.4093/kdj.2010.34.2.84 PMC2883355

[b57] Lu J. . Chronic dietary n-3 PUFA intervention improves dyslipidaemia and subsequent cardiovascular complications in the JCR:LA-cp rat model of the metabolic syndrome. British Journal of Nutrition 105, 1572–1582 (2011). 21276281 10.1017/S0007114510005453

[b58] Samimi M., Jamilian M., Asemi Z. & Esmaillzadeh A. Effects of omega-3 fatty acid supplementation on insulin metabolism and lipid profiles in gestational diabetes: Randomized, double-blind, placebo-controlled trial. Clin Nutr 34, 388–393 (2015). 24973862 10.1016/j.clnu.2014.06.005

[b59] Ostlund R. E. Jr. Phytosterols and cholesterol metabolism. Curr Opin Lipidol 15, 37–41 (2004). 15166807 10.1097/00041433-200402000-00008

[b60] Abumweis S. S., Barake R. & Jones P. J. Plant sterols/stanols as cholesterol lowering agents: A meta-analysis of randomized controlled trials. Food Nutr Res 52 (2008). 10.3402/fnr.v52i0.1811 PMC2596710 19109655

[b61] Micallef M. A. & Garg M. L. The lipid-lowering effects of phytosterols and (n-3) polyunsaturated fatty acids are synergistic and complementary in hyperlipidemic men and women. J Nutr 138, 1086–1090 (2008). 18492838 10.1093/jn/138.6.1086

[b62] Lyngdoh T. . Elevated serum uric acid is associated with high circulating inflammatory cytokines in the population-based Colaus study. PLoS One 6, e19901 (2011). 21625475 10.1371/journal.pone.0019901 PMC3098830

[b63] Netea M. G., Kullberg B. J., Blok W. L., Netea R. T. & van der Meer J. W. The role of hyperuricemia in the increased cytokine production after lipopolysaccharide challenge in neutropenic mice. Blood 89, 577–582 (1997). 9002961

[b64] Zapolski T. . Uric acid as a link between renal dysfunction and both pro-inflammatory and prothrombotic state in patients with metabolic syndrome and coronary artery disease. Kardiol Pol 69, 319–326 (2011). 21523662

[b65] Cheung B. M. & Li C. Diabetes and hypertension: is there a common metabolic pathway? Curr Atheroscler Rep 14, 160–166 (2012). 22281657 10.1007/s11883-012-0227-2 PMC3314178

[b66] Golbidi S. & Laher I. Exercise Induced Adipokine Changes and the Metabolic Syndrome. Journal of Diabetes Research 2014, 16 (2014). 10.1155/2014/726861 PMC3915640 24563869

[b67] Bellenger J. . High Pancreatic n-3 Fatty Acids Prevent STZ-Induced Diabetes in Fat-1 Mice: Inflammatory Pathway Inhibition. Diabetes 60, 1090–1099 (2011). 21330635 10.2337/db10-0901 PMC3064083

[b68] Malekshahi Moghadam A. . Efficacy of omega-3 fatty acid supplementation on serum levels of tumour necrosis factor-alpha, C-reactive protein and interleukin-2 in type 2 diabetes mellitus patients. Singapore Med J 53, 615–619 (2012). 23023905

[b69] Castaneda C. . Resistance training to reduce the malnutrition-inflammation complex syndrome of chronic kidney disease. Am J Kidney Dis 43, 607–616 (2004). 15042537 10.1053/j.ajkd.2003.12.025

[b70] Roberts C. K., Won D., Pruthi S., Lin S. S. & Barnard R. J. Effect of a diet and exercise intervention on oxidative stress, inflammation and monocyte adhesion in diabetic men. Diabetes Res Clin Pract 73, 249–259 (2006). 16616795 10.1016/j.diabres.2006.02.013

[b71] Ho R. C. . Regulation of IkappaB kinase and NF-kappaB in contracting adult rat skeletal muscle. Am J Physiol Cell Physiol 289, C794–801 (2005). 15888549 10.1152/ajpcell.00632.2004

[b72] Durham W. J. . Fatiguing exercise reduces DNA binding activity of NF-kappaB in skeletal muscle nuclei. J Appl Physiol (1985) 97, 1740–1745 (2004). 15208298 10.1152/japplphysiol.00088.2004

[b73] Newby A. C. Metalloproteinase expression in monocytes and macrophages and its relationship to atherosclerotic plaque instability. Arterioscler Thromb Vasc Biol 28, 2108–2114 (2008). 18772495 10.1161/ATVBAHA.108.173898

[b74] Lewandowski K. C., Banach E., Bienkiewicz M. & Lewinski A. Matrix metalloproteinases in type 2 diabetes and non-diabetic controls: effects of short-term and chronic hyperglycaemia. Arch Med Sci 7, 294–303 (2011). 22291770 10.5114/aoms.2011.22081 PMC3258712

[b75] Derosa G. . Evaluation of metalloproteinase 2 and 9 levels and their inhibitors in diabetic and healthy subjects. Diabetes Metab 33, 129–134 (2007). 17320450 10.1016/j.diabet.2006.11.008

[b76] Signorelli S. S. . Plasma levels and zymographic activities of matrix metalloproteinases 2 and 9 in type II diabetics with peripheral arterial disease. Vasc Med 10, 1–6 (2005). 15920993 10.1191/1358863x05vm582oa

[b77] Donley D. A. . Aerobic exercise training reduces arterial stiffness in metabolic syndrome. J Appl Physiol (1985) 116, 1396–1404 (2014). 24744384 10.1152/japplphysiol.00151.2014 PMC4044399

[b78] Kim J. S. . Effect of exercise training of different intensities on anti-inflammatory reaction in streptozotocin-induced diabetic rats. Biol Sport 31, 73–79 (2014). 25187675 10.5604/20831862.1093775 PMC3994589

[b79] Shinto L., Marracci G., Bumgarner L. & Yadav V. The Effects of Omega-3 Fatty Acids on Matrix Metalloproteinase-9 Production and Cell Migration in Human Immune Cells: Implications for Multiple Sclerosis. Autoimmune Diseases 2011 (2011). 10.4061/2011/134592 PMC3140187 21799946

[b80] Harris M. A. . Effects of conjugated linoleic acids and docosahexaenoic acid on rat liver and reproductive tissue fatty acids, prostaglandins and matrix metalloproteinase production. Prostaglandins Leukot Essent Fatty Acids 65, 23–29 (2001). 11487304 10.1054/plef.2001.0283

[b81] Gupta S., Singh R. K., Dastidar S. & Ray A. Cysteine cathepsin S as an immunomodulatory target: present and future trends. Expert Opin Ther Targets 12, 291–299 (2008). 18269339 10.1517/14728222.12.3.291

[b82] Liu J. . Increased serum cathepsin S in patients with atherosclerosis and diabetes. Atherosclerosis 186, 411–419 (2006). 16140306 10.1016/j.atherosclerosis.2005.08.001

[b83] Naour N. . Cathepsins in human obesity: changes in energy balance predominantly affect cathepsin s in adipose tissue and in circulation. J Clin Endocrinol Metab 95, 1861–1868 (2010). 20164293 10.1210/jc.2009-1894

[b84] Taleb S. . Weight loss reduces adipose tissue cathepsin S and its circulating levels in morbidly obese women. J Clin Endocrinol Metab 91, 1042–1047 (2006). 16394095 10.1210/jc.2005-1601

[b85] Taleb S. . Cathepsin S, a novel biomarker of adiposity: relevance to atherogenesis. FASEB J 19, 1540–1542 (2005). 15985526 10.1096/fj.05-3673fje

[b86] Jobs E. . Influence of a prudent diet on circulating cathepsin S in humans. Nutr J 13, 84 (2014). 25128296 10.1186/1475-2891-13-84 PMC4155124

[b87] Farilla L. . Glucagon-like peptide-1 promotes islet cell growth and inhibits apoptosis in Zucker diabetic rats. Endocrinology 143, 4397–4408 (2002). 12399437 10.1210/en.2002-220405

[b88] Coughlan K. A., Valentine R. J., Ruderman N. B. & Saha A. K. AMPK activation: a therapeutic target for type 2 diabetes? Diabetes Metab Syndr Obes 7, 241–253 (2014). 25018645 10.2147/DMSO.S43731 PMC4075959

[b89] Svegliati-Baroni G. . Glucagon-like peptide-1 receptor activation stimulates hepatic lipid oxidation and restores hepatic signalling alteration induced by a high-fat diet in nonalcoholic steatohepatitis. Liver Int 31, 1285–1297 (2011). 21745271 10.1111/j.1478-3231.2011.02462.x

[b90] Viollet B. . Targeting the AMPK pathway for the treatment of Type 2 diabetes. Front Biosci (Landmark Ed) 14, 3380–3400 (2009). 19273282 10.2741/3460 PMC2677695

[b91] Salminen A., Hyttinen J. M. & Kaarniranta K. AMP-activated protein kinase inhibits NF-kappaB signaling and inflammation: impact on healthspan and lifespan. J Mol Med (Berl) 89, 667–676 (2011). 21431325 10.1007/s00109-011-0748-0 PMC3111671

[b92] Flynn M. G., McFarlin B. K. & Markofski M. M. The Anti-Inflammatory Actions of Exercise Training. Am J Lifestyle Med 1, 220–235 (2007). 25431545 10.1177/1559827607300283 PMC4243532

[b93] Schultze S. M., Hemmings B. A., Niessen M. & Tschopp O. PI3K/AKT, MAPK and AMPK signalling: protein kinases in glucose homeostasis. Expert Rev Mol Med 14, e1 (2012). 22233681 10.1017/S1462399411002109

[b94] Katerelos M. . 5-aminoimidazole-4-carboxamide ribonucleoside and AMP-activated protein kinase inhibit signalling through NF-kappaB. Immunol Cell Biol 88, 754–760 (2010). 20404837 10.1038/icb.2010.44

[b95] Huang N. L. . Metformin inhibits TNF-alpha-induced IkappaB kinase phosphorylation, IkappaB-alpha degradation and IL-6 production in endothelial cells through PI3K-dependent AMPK phosphorylation. Int J Cardiol 134, 169–175 (2009). 18597869 10.1016/j.ijcard.2008.04.010

[b96] Higuchi T., Shirai N., Saito M., Suzuki H. & Kagawa Y. Levels of plasma insulin, leptin and adiponectin, and activities of key enzymes in carbohydrate metabolism in skeletal muscle and liver in fasted ICR mice fed dietary n-3 polyunsaturated fatty acids. The Journal of Nutritional Biochemistry 19, 577–586 (2008). 17911005 10.1016/j.jnutbio.2007.08.001

