# Supplementary material for: Purslane (Portulaca oleracea) Seed Consumption And Aerobic Training Improves Biomarkers Associated with Atherosclerosis in Women with Type 2 Diabetes (T2D)
Source: Sci Rep. 2016 Dec 5;6:37819. doi: 10.1038/srep37819 (PMC5137030; doi:10.1038/srep37819)
Supplement: Supplementary Information [file srep37819-s1.docx]

**Supplementary file**

**Purslane (Portulaca oleracea) Seed Consumption And Aerobic Training Improves Biomarkers Associated with Atherosclerosis in Women with Type 2 Diabetes (T2D)**

Firouzeh Dehghan*^1^, Rahman Soori*^1^, Khadijeh Gholami^2^, Mitra Abolmaesoomi^3^, Ashril Yusof^4^, Sekaran Muniandy^3^, Sara Heidarzadeh^5^, Parvin Farzanegi^6^, Mohammad Ali azarbayjani^5^

^1^Department of Exercise Physiology, Faculty of Physical Education and Sport Sciences, University of Tehran, Tehran, Iran

^2^Department of Physiology, Faculty of Medicine, University of Malaya, 50603 Kuala Lumpur, Malaysia

^3^Department of Molecular Medicine, Faculty of Medicine, University of Malaya, 50603 Kuala Lumpur, Malaysia

^4^Department of Exercise Science, Sports Centre, University of Malaya, 50603 Kuala Lumpur, Malaysia

^5^Department of Exercise Physiology, Central Tehran Branch, Islamic Azad University, Tehran, Iran

^6^Department of Exercise Physiology, Sari Branch, Islamic Azad University, Sari, Iran.

* Corresponding authors:

Firouzeh Dehghan; Department of Exercise Physiology, Faculty of Physical Education and Sport Sciences, University of Tehran, Tehran, Iran

Tel: +9821 61118875, Fax: +9821 88021527 Email: [firouzeh.dehghan@ut.ac.ir](mailto:firouzeh.dehghan@ut.ac.ir)

Rahman Soori; Department of Exercise Physiology, Faculty of Physical Education and Sport Sciences, University of Tehran, Tehran, Iran

Tel: +9821 61118875, Fax: +9821 88021527 Email: [soori@ut.ac.ir](mailto:soori@ut.ac.ir)

Chromatography/Mass Spectroscopy (GC/MS/MS)

The extract was diluted in GC grade methanol (1 mL) and analyzed with Agilent Technologies 7890A with LECO PENGASUS HT High Throughput TOFMS with a Rxi-5MS capillary column, with a (5% diphenyl)- 95% polymethylsiloxane stationary phase, film thickness of 0.25μm, a length of 30 m, and an internal diameter of 0.25 mm was used for separation. Helium was used as a carrier gas with a flow rate of 1.0 mL/min; detector’s temperature was 280°C, and interface temperature was 250°C. Samples (1 μL) were injected into the injector with a splitless mode. The GC oven temperature program was used as follows: 70°C initial temperature, hold for 2 min and increased at 5°C/min to 290°C, hold for 5 min and the total run time was approximately 51 minutes. Mass spectra: electron impact (EI+) mode, 70ev and ion source temperature 200°C. Mass spectra were recorded over 50-1000 a.m.u range. Identification of the compounds was based on comparison of the corresponding mass spectra with data from the MS library [mainlib, replib and HPCH2205]. The relative amount (RA) of each oil component is expressed as percent peak area relative to the total peak area.

ELISA kits information detail:

Enzyme-linked immunosorbent assay (ELISA) was performed by using commercial kits (CUSABIO - USA) for NF-κB with detection range of 0.312-20ng/mL and sensitivity of 0.078ng/mL; GLP-1 with detection range of 2.29-40ng/ml and sensitivity of 1.45ng/ml; GLP1R with detection range of 0.156 ng/ml-10ng/ml and sensitivity of 0.039ng/ml; TIMP-1, with detection range of 0.39-25ng/ml and sensitivity of 0.434ng/ml; MMP2, with detection range of 0.78-50ng/ml and sensitivity of 0.195ng/ml; MMP9, with detection range of 0.312-20ng/ml and sensitivity of 0.284 ng/ml; CRP with detection range of 0.625-40ng/ml and sensitivity of 0.156ng/ml; CST3 with detection range of 7.8-500ng/ml and sensitivity of 5.824ng/ml; CTSS with detection range of 1.25-80ng/ml and sensitivity of 0.31ng/ml.

Primer assays information detail:

NF-kB (nuclear factor of kappa light polypeptide gene enhancer in beta-cells) with assay id: Hs00765730_m1; Lot no: 4331182; and amplicon length of 66. GLP-1 (glucagon-like peptide 1) with assay id: Hs01031536_m1; Lot no: 4331182; and amplicon length of 86. GLP1-R (glucagon-like peptide 1 receptor) with assay id: Hs01006326_m1; Lot no: 4351372; and amplicon length of 98. TIMP-1 (metallopeptidase inhibitor 1) with assay id: Hs00171558_m1; Lot no: 4331182; and amplicon length of 104. MMP2 (matrix metallopeptidase 2) with assay id: Hs01548727_m1; Lot no: 4331182; and amplicon length of 65. MMP9 (matrix metallopeptidase 9) with assay id: Hs00234579_m1; Lot no: 4331182; and amplicon length of 79. CRP (C-reactive protein, pentraxin-related) with assay id: Hs04183452_g1; Lot no: 4331182; and amplicon length of 99. CST3 (Cystatin C) with assay id: Hs00969174_m1; Lot no: 4331182; and amplicon length of 63. CTSS (Cathepsin S) with assay id: Hs00175407_m1; Lot no: 4331182; and amplicon length of 70. ACTB (actin, beta) which was used as reference gene with assay id: Hs01060665_g1; Lot no: 4331182; and amplicon length of 63.
